# Hemifacial Spasm as the Presenting Manifestation of Type 3c Diabetes Mellitus

**DOI:** 10.5334/tohm.611

**Published:** 2021-04-26

**Authors:** Ritwik Ghosh, Dipayan Roy, Subhankar Chatterjee, Souvik Dubey, Bikash Chandra Swaika, Arpan Mandal, Julián Benito-León

**Affiliations:** 1 Department of General Medicine, Burdwan Medical College & Hospital, Burdwan, West Bengal, India; 2 Department of Biochemistry, All India Institute of Medical Sciences (AIIMS), Jodhpur, Rajasthan, India; 3 Indian Institute of Technology (IIT), Madras, Tamil Nadu, India; 4 Department of General Medicine, Patliputra Medical College & Hospital, Dhanbad, Jharkhand, India; 5 Department of Neuromedicine, Bangur Institute of Neurosciences (BIN), Kolkata, West Bengal, India; 6 Department of Neurology, University Hospital “12 de Octubre”, Madrid, Spain; 7 Centro de Investigación Biomédica en Red sobre Enfermedades Neurodegenerativas (CIBERNED), Madrid, Spain; 8 Department of Medicine, Complutense University, Madrid, Spain

**Keywords:** pancreatogenic diabetes, type 3c diabetes, hemifacial spasm, chronic pancreatitis

## Abstract

**Background:**

Type 3c diabetes mellitus (T3cDM) usually occurs because of a variety of exocrine pancreatic diseases with varying mechanisms, which eventually lead to secondary pancreatic endocrine insufficiency i.e. hyperglycemia.

**Phenomenology:**

A man suffering from previously undiagnosed T3cDM presenting with subacute onset hemifacial spasm.

**Educational value:**

This case emphasizes the importance of rapid bedside measurement of capillary blood glucose in patients presenting with acute to subacute onset movements disorders irrespective of their past glycemic status.

## Introduction

Type 3c diabetes mellitus (T3cDM), or pancreatogenic diabetes mellitus, may occur because of a variety of exocrine pancreatic diseases, mainly acute or chronic pancreatitis, pancreatic trauma, malignancy, hemochromatosis, and cystic fibrosis [1]. Among these, chronic pancreatitis is the most common cause [2]. The pathophysiology includes impaired hormonal regulation of glucose homeostasis and activation of hepatic gluconeogenesis, leading to glycemic instability [1]. We herein report a case of a 31-year-old patient, with a history of chronic pancreatitis, who presented with hemifacial spasm. The patient was found to have hyperglycemia and eventually diagnosed as T3cDM, which responded remarkably to insulin.

### Case presentation

A 31-year-old non-smoker and non-alcoholic man presented to the emergency department with abdominal pain. He also complained of intermittent twitching surrounding his eyelids for the last three months, which had aggravated recently for the last 12 days. He had a history of pancreatitis six years ago, which had been managed conservatively. He did not visit for any further follow-up, as he was asymptomatic except involuntary weight loss and passage of foul-smelling oily diarrhea for last 1 month, suggestive of steatorrhea. Family history and drug history were non-contributory. The patient was afebrile and normotensive. On physical examination, intermittent twitching involving muscles on the right side of the face, including the eyelid, was observed. (***Video***).

**Video:** Video showing hemifacial spasm involving muscles on the right side of the face, including the eyelid.

The arterial blood gas analysis, serum ketones and serum osmolarity were within normal limits. C-peptide levels were low (0.4 ng/mL). Complete blood cell count, renal, hepatic and thyroid function tests, and lipid profile, were within normal limits. A magnetic resonance imaging of the brain as well as a magnetic resonance angiography revealed no significant findings, thus ruling out any structural, cerebrovascular, metabolic or demyelinating etiology.

Relevant tests for detection of pancreatic exocrine insufficiency were positive. Pancreatic islet cells and glutamic acid decarboxylase (GAD)-65 autoantibodies were negative. Antinuclear antibody, antineutrophil cytoplasmic antibody, HIV, VDRL, and hepatitis C virus serology were also negative. The absence of hilar lymphadenopathy as well as normal serum levels of angiotensin-converting enzyme and calcium ruled out neurosarcoidosis. A multi-detector computed tomography scan of the abdomen revealed pancreatic atrophy, duct dilatations and calcifications, suggestive of chronic pancreatitis. A bedside capillary blood glucose measurement revealed hyperglycemia (332 mg/dL). Fasting and post-prandial blood glucose were 210 mg/dL and 320 mg/dL, respectively. HbA1c was estimated to be 8.6%. A diagnosis of pancreatogenic diabetes mellitus (type 3c) was made on the backdrop of chronic pancreatitis. The patient was treated with premixed insulin [Insulin isophane/NPH (70%) and human insulin/soluble insulin (30%)]. Hemifacial spasm disappeared with achieving euglycemia and did not recur.

## Discussion

T3cDM is a relatively new entity on which the medical literature is scarce. The proposed major criteria for diagnosing T3cDM include: a) exocrine pancreatic inefficiency; b) pancreatic pathology observed in imaging; and c) absence of type 1 diabetes mellitus-associated autoantibodies [3]. Our patient met all of them.

Acute to subacute onset movement disorders (mostly chorea and ballism) as the presenting feature of non-ketotic and ketotic hyperglycemic complications (mostly among type-2 diabetics) are well recognized in the literature [4,5]. With simple correction of the hyperglycemic state, these movements usually subside [4,5]. However, hyperglycemia-associated hemifacial spasm in T3cDM has not been previously reported.

Pathogenetic mechanisms for the development of movement disorders in T3cDM may be either mediated by hyperglycemia-induced vasa nervorum injury, leading to facial nerve damage (part of diabetic cranial neuropathy), or by direct neurotoxic effect of hyperglycemia [6]. The neuronal damage may be hastened by non-enzymic glycosylation of structural proteins (e.g. laminin), free radical damage, increased concentration of pro-inflammatory markers (e.g. tumor necrosis factor-alpha, interleukins), and lack of neurotrophins, such as insulin-like growth factor (IGF-1 and IGF-2) and nerve growth factor [7]. Furthermore, anatomical variations and myelination patterns may play a role in the vulnerability of certain cranial nerves in diabetes.

Ours is a unique case where hemifacial spasm was detected in the setting of previously undiagnosed T3cDM. Moreover, hemifacial spasm disappeared following the establishment of a normoglycemic state, thus emphasizing the importance of correction of hyperglycemia. It further helps in arriving at the diagnosis much earlier and avoiding potential mismanagement. Ultimately, this case emphasizes the importance of checking the glycemic status of patients presenting with movement disorders, and therefore, has practical implications for clinicians.
